# Hôtel Dieu

**Published:** 1831-06

**Authors:** 


### HÔTEL DIEU.

### *Inflammation of a Hernial Sac, with Symptoms of Strangulation: Operation.*

A WOMAN, fifty years of age, was admitted into the Hôtel Dieu, who had had crural hernia of the right side for many years. A bandage had been properly applied, and constantly worn. For some time, and without any known cause, the groin and the part upon which the bandage pressed became painful. The patient complained of colic, constipation, and frequent attempts to vomit. When she was admitted, the tumor was painful to the touch, of a globular form, and about the size of a large pigeon's egg: it was hard and irreducible; the colour of the skin was unchanged. These symptoms had existed for some days, in addition to which there were now occasional hiccup and vomiting. It was presumed that a portion of intestine was strangulated. Bleeding, warm baths, emollient applications, &c. were first tried; the taxis also was several times had recourse to, without success.

M. SANSON, finding that the symptoms still continued, determined to operate without further delay. An incision was made upon the tumor, as in the operation for hernia, and the surgeon then very cautiously proceeded to divide the parts above the hernial sac, with a bistoury and grooved director. When he had arrived at the sac, a puncture was made into it, and some pus escaped. The puncture was enlarged, and all the purulent fluid contained in the tumor was evacuated. The operator introduced his finger, but could discover neither omentum nor intestine. The internal surface of the hernial sac was smooth, polished, and free from adhesions, and at the upper part there was the orifice of a very narrow canal, which communicated with the abdomen: this canal was so small, that the end of the little finger could with difficulty be introduced into it: it was the remains of the neck of the old hernial sac. Immediately after the operation, all the symptoms, which so strongly resembled those which arise from strangulation of a portion of intestine or omentum, entirely ceased.

